# Characterization of the upstream and intron promoters of the gene encoding TAR DNA-binding protein

**DOI:** 10.1038/s41598-021-88015-y

**Published:** 2021-04-22

**Authors:** Minami Hasegawa-Ogawa, Hirotaka James Okano

**Affiliations:** Division of Regenerative Medicine, The Jikei University School of Medicine, 3-25-8 Nishi-Shimbashi, Minato-ku, Tokyo, 1058461 Japan

**Keywords:** Biochemistry, Cell biology, Genetics, Molecular biology

## Abstract

TAR DNA-binding protein (TDP-43, encoded by *TARDBP*) is a multifunctional protein that regulates transcription and RNA metabolism by binding DNA or RNA. TDP-43 has been implicated in the pathogenesis of amyotrophic lateral sclerosis (ALS) because abnormal accumulation of cleaved and phosphorylated C-terminal fragments of TDP-43 in motor neurons is a pathological hallmark of ALS. Here, we cloned and analyzed the promoter region of the *TARDBP* gene. *TARDBP* upstream sequences and/or intron/luciferase constructs were generated, and their promoter activity was experimentally assessed. The upstream region predictably exhibited promoter activity and identified putative *cis*-acting elements, including the i-motif*,* was relevant for the regulation of TDP-43 expression. The cellular abundance of TDP-43 is strictly controlled, and its constancy is critically important for motor neuron survival. A machinery serving to maintain a constant level of TDP-43 is autoregulation via control of mRNA stability, a negative feedback system involving binding to the 3′ untranslated region of its own pre-mRNA. However, whether transcriptional mechanisms contribute to TDP-43 autoregulation is unclear. We further showed that TDP-43 negatively regulates the *TARDBP* promoter and, surprisingly, that disease-causing TDP-43 mutants lacked this regulatory activity. These results allowed the elucidation of a novel transcriptional autoregulatory mechanism of TDP-43.

## Introduction

Amyotrophic lateral sclerosis (ALS) is a neurodegenerative disease that selectively affects motor neurons (MNs) in the brain motor cortex and spinal cord and is characterized by late-onset progressive muscle weakness and atrophy, and respiratory failure, leading to death typically within several years^1, 2^. Approximately 5–10% of ALS cases are familial (familial ALS, fALS), and more than 20 causative genes have been identified from fALS, such as superoxide dismutase 1 (SOD1), TAR DNA-binding protein 43 kDa (TDP-43), fused in sarcoma (FUS), optineurin and chromosome 9 open reading frame 72 (C9orf72)^3–16^. Some causative gene mutations are found not only in fALS but also in sporadic ALS (sALS). In fact, more than 30 disease-related mutations of TDP-43 have been found, and some TDP-43 mutations are found in both fALS and sALS^5–11^. A characteristic disease hallmark in ALS pathology is the frequent presence of insoluble ubiquitin-positive inclusions in MNs of ALS patients^1, 2^. The neuronal inclusions located in the cytoplasm or nucleus include abnormally cleaved and phosphorylated C-terminal fragments of TDP-43^17–23^. In recent years, TDP-43 was shown to be present in the inclusions of more than 97% of ALS patients, including those with fALS and sALS, even if the patient did not harbor TDP-43 mutations^24^. Although whether TDP-43 affects MNs by loss of function or gain of toxic function remains controversial, these findings imply that TDP-43 plays a critical role in ALS onset and progression.

TDP-43, encoded by *TARDBP*, was initially identified as a transcriptional regulator that binds to a regulatory element in the human immunodeficiency virus type 1 (HIV-1) long terminal repeat known as TAR and suppresses HIV-1 gene expression^25^. TDP-43 binds not only DNA but also RNA, thus regulating aspects of RNA metabolism such as alternative splicing and mRNA stability^24, 26–28^.TDP-43 includes two RNA recognition motifs (RRM) and binds to DNA/RNA targets. It is known that RRM1 is necessary and sufficient for DNA/RNA binding activity^26^.

In organisms, tight control of TDP-43 expression is very important. TDP-43 gene knockout (KO) was embryonically lethal in mice at E3.5–12.5, and even heterozygotic animals appeared to develop motor impairment late in life^29^. In addition, MN-specific TDP-43 conditional KO mice developed motor dysfunctions such as abnormal clasping, decreased time on a rotarod, and late-onset loss of MNs^30^. These findings indicate that TDP-43 is essential for ontogeny and necessary for the survival of MNs. Furthermore, regarding the TDP-43 overexpression model, PrP promoter-mutant human TDP-43 transgenic (Tg) mice developed weak grip strength and spinal MN loss at 10 months of age^31^. Similarly, Thy-1 promoter-human wild-type (WT) TDP-43 Tg mice, in which the transgene is expressed virtually all neurons in the central nervous system (CNS), showed an abnormal hindlimb reflex response, MN loss and ubiquitin-positive aggregates that were also positive for phosphorylated TDP-43 in spinal MNs^32^. These findings indicate that excessive expression of mutant as well as WT TDP-43 leads to MN degeneration. Thus, inappropriate TDP-43 levels affect the homeostasis of MNs, and the level of TDP-43 should be strictly regulated within the appropriate range. A recent study showed that the envelope protein of human endogenous retrovirus K (HERV-K) is expressed in the motor cortex and spinal cord of patients with sALS but not in healthy controls^33^. Interestingly, TDP-43 interacts with consensus sequences in the HERV-K promoter and regulates its transcription. From these findings, although TDP-43 is often characterized as a regulator of RNA metabolism, TDP-43 as a transcriptional regulator may also have an important role in ALS pathophysiology.

Previous reports have shown that the TDP-43 level is strictly regulated by a negative feedback mechanism via an alternative polyA site in the 3′ untranslated region (UTR)^23, 34, 35^ of TDP-43 mRNA; however, whether the TDP-43 promoter is also involved in autoregulatory systems is unknown. The predicted TDP-43 promoter region was identified previously by using a promoter prediction database^36^, but no experimental study has been performed. In this study, we experimentally investigated whether the *TARDBP* upstream region shows promoter activity and demonstrated that the *TARDBP* promoter is involved in TDP-43 autoregulation.

## Results

### The *TARDBP* upstream region exhibits promoter activity

**Figure 1:**
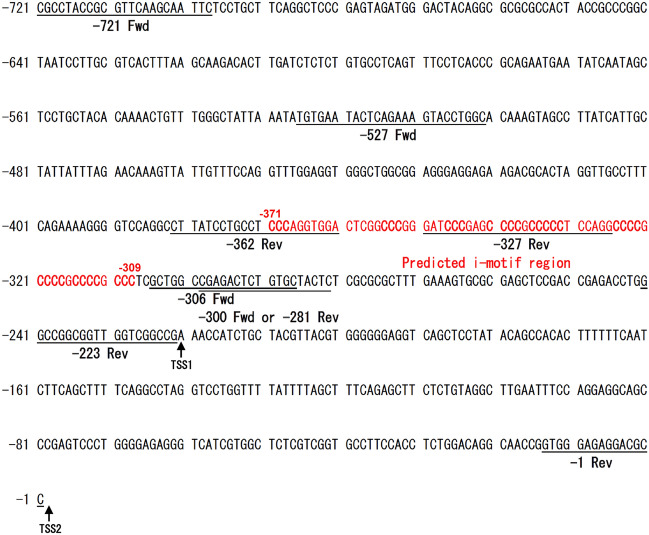
*TARDBP* upstream sequences from the UCSC genome browser. The 721 nucleotides in the *TARDBP* upstream sequence include the predicted promoter region shown in a previous study. The red characters indicate the predicted i-motif region. Cytosine-rich regions are denoted by bolded text. The primer sequences used to generate the promoter/reporter constructs are underlined. *Fwd* forward primer, *Rev* reverse primer, *TSS* transcription start site. The UCSC genome browser shows 2 TSSs, and TSS1 is located 223 bp upstream of TSS2.

**Figure 2:**
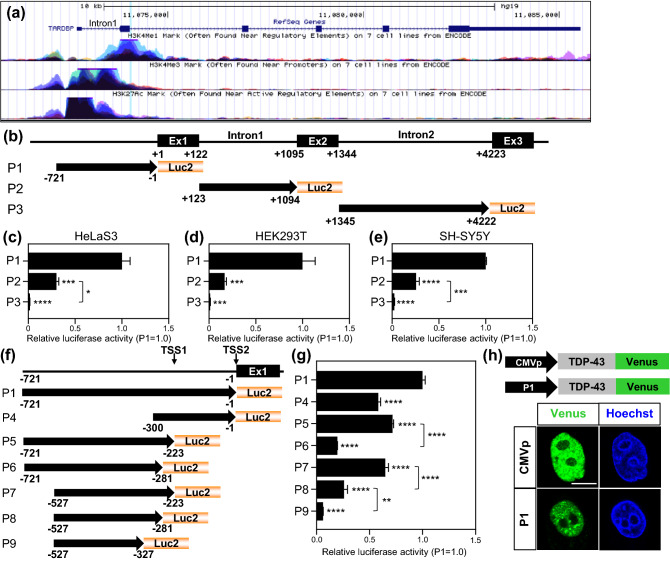
The *TARDBP* upstream region exhibits promoter activity. (**a**) Epigenetic marker activity in the human *TARDBP* region in 7 cell lines from ENCODE is exhibited by the UCSC genome browser. H3K4me1 (often found near regulatory elements), H3K4me3 (often found near promoters) and H3K27ac (often found near active regulatory elements) are described. The *TARDBP* genomic position is chr1:11072679–11085549 in GRCh37/hg19, and the gene accession code from NCBI is NM_007375. (**b**) Schematic representation of the *TARDBP* upstream promoter and intron/Luc2 constructs (P1–3). The black boxes and lines indicate exons (Ex) and introns, respectively. + 1 indicates TSS2 (see Fig. 1). P1 (− 721/− 1), P2 (+ 123/ + 1094, intron 1), and P3 (+ 1345/ + 4222, intron 2). (**c**–**e**) *TARDBP* promoter/luciferase assay in 3 cell lines: HeLaS3 (**c**), HEK293T (**d**) and SH-SY5Y (**e**). n = 3 replicates; one-way ANOVA with post hoc Tukey’s test relative to P1. (**f**) Schematic representation of *TARDBP* promoter constructs (P4–9). P4 (− 300/− 1), P5 (− 721/− 223), P6 (− 721/− 281), P7 (− 527/− 223), P8 (− 527/− 281), and P9 (− 527/− 327). (**g**) Luciferase assay of *TARDBP* promoter fragments. Quantitative analyses have n = 9 (P1), n = 6 (P7) and n = 3 (P4, P5, P6, P8 and P9) replicates. One-way ANOVA with post hoc Tukey’s test was used, and all error bars indicate the means ± SEMs. **P < 0.01, ***P < 0.001, ****P < 0.0001. (**h**) Fluorescence images of TDP-43-Venus (Green) expression driven by the CMV promoter (CMVp) or *TARDBP* upstream promoter (P1) in HeLaS3 cells. Nuclei were stained with Hoechst (blue). Scale bar 10 μm.

Predicted promoter regions of *TARDBP* were determined by the programs ElDorado and Promoter Scan in a previous study^36^. We cloned the *TARDBP* upstream sequence starting at nucleotide − 721, which included the predicted promoter region and the sequences described in Fig. 1. We also predicted the *TARDBP* promoter region using the UCSC genome browser. In agreement with the previous prediction, the region upstream of the transcription start site (TSS), exon 1 and intron 1 exhibited high enrichment with epigenetic markers of active transcription (Fig. 2a). Therefore, *TARDBP* upstream or intron/luciferase (encoded by Luc2) reporters containing the predicted region were generated on pcDNA3.1(−) backbone, and their promoter activities were determined (Fig. 2b). Promoter construct 1 (P1) includes the *TARDBP* upstream sequence from nucleotide -721 relative to the TSS, while P2 and P3 include intron 1 and intron 2, respectively. P3 was used as the negative control. Promoter activity was demonstrated by a luciferase assay in three cell lines—human cervical cancer HeLaS3, embryonic kidney HEK293T and neuroblastoma SH-SY5Y (Fig. 2c–e). P1 showed a marked luciferase signal compared with that of P3 in all cell lines; the P1 signal was increased relative to that of P3 by 58.8-, 76.9- and 41.7-fold in HeLaS3, HEK293T and SH-SY5Y cells, respectively. Interestingly, P2 also exhibited a greater level of promoter activity than P3: increases of 17.8-fold in HeLaS3 cells, 12.8-fold in HEK293T cells and 10.7-fold in SH-SY5Y cells. To identify a functional promoter element, the additional reporters P4-9 were generated: P4 (− 300/− 1), P5 (− 721/− 223), P6 (− 721/− 281), P7 (− 527/− 223), P8 (− 527/− 281) and P9 (− 527/− 327) (Fig. 2f). P4 exhibited a 41.8% decrease in activity relative to P1, suggesting that the region between nucleotides − 721 and − 300 includes an important element for full promoter activity (Fig. 2g). In the analysis of *TARDBP* upstream sequences, the UCSC genome browser showed two TSSs (TSS1 and TSS2; Fig. 2f) based on accession no. NM_007375 from NCBI. P5, which includes the upstream sequence of TSS1, showed a 28.3% decrease in activity relative to P1. Interestingly, P6, which has a deletion of 58 nucleotides from the 3′ end of P5, resulted in a marked reduction (80.3% decrease) in promoter activity, suggesting that the region including the proximal TSS (TSS1) is crucial in the promoter. When 104 nucleotides in the 3′ end of P7 were removed (forming P9), a significant decrease in promoter activity was observed (93.8% decrease). Our data suggested that both proximal and distal upstream sequences are necessary for maximal promoter activity and that important *cis*-acting elements for regulatory factors may be present in the region between nucleotide − 327 and TSS1. Furthermore, to determine whether P1 can drive TDP-43 expression, constructs harboring TDP-43-Venus with the cytomegalovirus promoter (CMVp) or with P1 were transfected into HeLaS3 cells. Although the level of TDP-43-Venus expression driven by P1 was lower than that driven by the CMV promoter, a moderate level of TDP-43-Venus expression was driven by P1 (Fig. 2h).

### The *TARDBP* intron exhibits promoter activity

**Figure 3:**
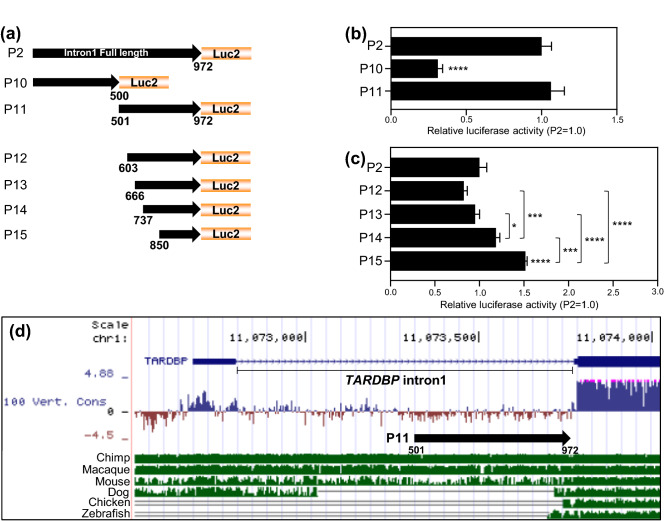
*TARDBP* intron 1 shows weak promoter activity. (**a**) Schematic images of human *TARDBP* intron 1/luciferase reporter (P2, 10–15) constructs. Each fragment name is described below: P2 (1/972, full length of intron 1), P10 (1/500), P11 (501/972), P12 (603/972), P13 (666/972), P14 (737/972), and P15 (850/972). (**b**,**c**) Luciferase assay of *TARDBP* introns 1/luciferase reporter constructs. n = 5 (**b**) and n = 7 (**c**) replicates; one-way ANOVA with post hoc Dunnett’s test relative to P2 (**b**) or Tukey’s test (**c**). All error bars indicate the means ± SEMs. *P < 0.05, ***P < 0.001, ****P < 0.0001. (**d**) Analysis of *TARDBP* intron 1 sequence conservation among the chimpanzee, macaque, mouse, dog, chicken and zebrafish genomes using the UCSC genome browser. The thick black arrow indicates the proximal half of intron 1, P11 (501/972).

The P2 (full length of intron 1) reporter construct showed promoter activity levels that were 30.2% (HeLaS3), 16.7% (HEK293T), and 25.7% (SH-SY5Y) that of P1 (Fig. 2c–e). Then, we generated various intron 1/luciferase promoter constructs to identify the element responsible for promoter activity: P2 (1/972), P10 (1/500), P11 (501/972), P12 (603/972), P13 (666/972), P14 (737/972) and P15 (850/972) (Fig. 3a). The distal P10 (1/500) fragment exhibited a 68.9% decrease in luciferase activity relative to P2. In contrast, the promoter activity of the proximal P11 (501/972) fragment was similar to that of P2, suggesting that the proximal half of intron 1 is required for promoter activation (Fig. 3b). To further characterize the core promoter region, an additional 4 intron 1 fragments—P12 (603/972), P13 (666/972), P14 (737/972) and P15 (850/972)—were analyzed (Fig. 3a,c). The luciferase activity of the shortest fragment (P15) was significantly higher than that of the other fragments, indicating that P15 includes the core activation region in this promoter. However, the 912/972 fragment did not exhibit any promoter activity (data not shown). In addition, our results suggest that the core activation region in the intron 1 promoter may be regulated by the suppressor element in the region comprising nucleotides 666–850, since the activity of P15 was significantly higher than that of P13 or P14. As the proximal half of the intron 1 promoter seems to have physiological significance, we analyzed the conservation of the intron 1 sequence in vertebrates via the UCSC genome browser. Surprisingly, the proximal half of intron 1 is conserved only among primates such as macaques and chimpanzees but not mice, and it is deleted from the dog genome (Fig. 3d). *TARDBP* intron 1 sequences are described in Supplementary Figure S1.

**Figure 4:**
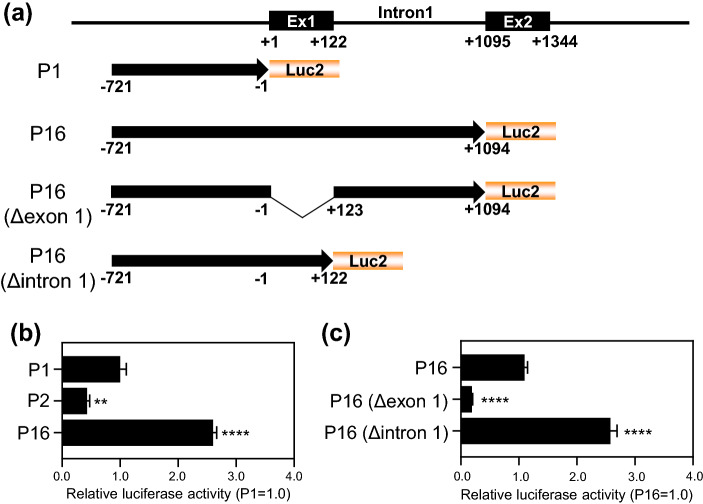
*TARDBP* exon 1 is crucial for upstream promoter activity. (**a**) Schematic representation of P16 (− 721/+ 1094), P16 (Δexon 1) and P16 (Δintron 1). (**b**) Luciferase assay of P1 (upstream promoter), P2 (intron 1 promoter) and P16. n = 3 replicates; one-way ANOVA with post hoc Dunnett’s test relative to P1. (**c**) Luciferase assay of P16, P16 (Δexon 1) and P16 (Δintron 1). n = 3 replicates; one-way ANOVA with post hoc Dunnett’s test relative to P16. All error bars indicate the means ± SEMs. **P < 0.01, ****P < 0.0001.

To determine whether the noncoding exon 1 and intron 1 sequences in *TARDBP* are crucial for the activation of the upstream promoter, a reporter construct harboring an upstream promoter together with exon 1 and intron 1 was generated: P16 (− 721/+ 1094) (Fig. 4a). Relative to P1, P16 exhibited a marked 2.6-fold increase in promoter activity (Fig. 4b). To further study the activity of P16, P16-related constructs were generated: P16 (Δexon 1) and P16 (Δintron 1) (Fig. 4a). P16 (Δexon 1) exhibited an 80.5% decrease in luciferase activity, in addition, P16 (Δintron 1) showed 2.6-fold increase in promoter activity relative to P16 (Fig. 4c). RT-PCR analysis revealed that the intron 1 of the P16 was spliced out and the spliced transcript with exon 1 was produced which was the same transcript generated with the P16 (Δintron 1) (Fig. S2a). These results indicate that the intron 1 sequences are not crucial for the *TARDBP* promoter and suggest that exon 1 plays an important role for TDP-43 expression probably by posttranscriptional mechanisms.

### TDP-43 inhibits the *TARDBP* promoter

**Figure 5:**
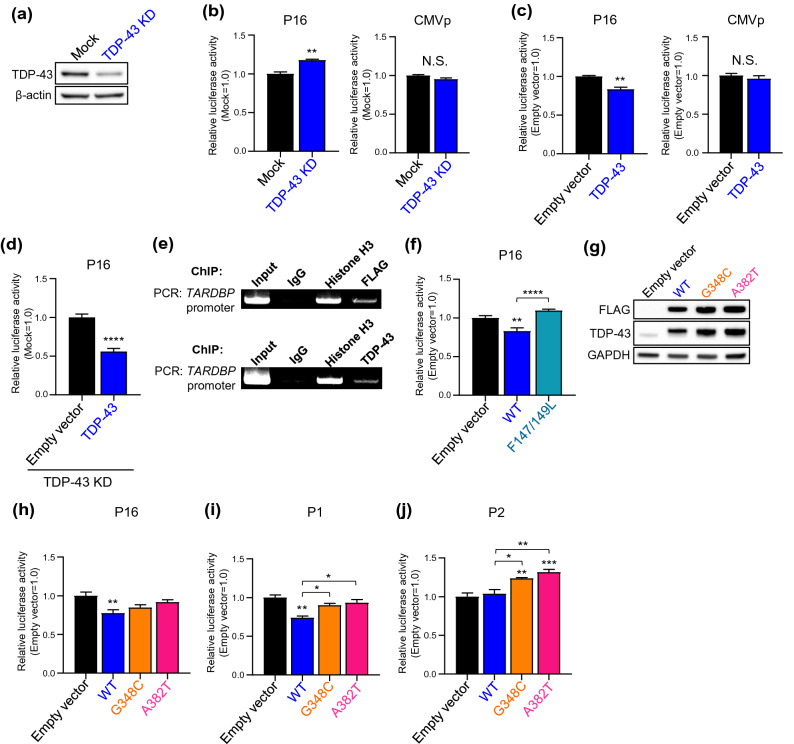
TDP-43 regulates the activity of the *TARDBP* upstream and intron 1 promoter. (**a**) Immunoblot analysis of TDP-43 in HeLaS3 cells. Mock and KD indicate transfected negative control or TDP-43 siRNA, respectively. (**b-d**) Luciferase assay of P16 or CMV promoter activity with TDP-43 KD (**b**) or overexpression (**c**) or both endogenous TDP-43 KD and exogenous TDP-43 overexpression (**d**). n = 4 (**b, c**) and n = 8 (**d**) replicates; Welch’s t-test relative to Mock (**b**) or Empty vector (**c**,**d**). (**e**) ChIP assay of overexpressed FLAG-TDP-43 (*top*) or endogenous TDP-43 (*bottom*) binding to the *TARDBP* promoter in HEK293T cells. Immunoprecipitation was performed using anti-DDDDK (FLAG) antibody (for overexpressed FLAG-TDP-43) or anti-TDP-43 antibody (for endogenous TDP-43), then PCR was conducted using primer detecting *TARDBP* -721 to -431 (290 bp). Control IgG and Histone H3 antibody are used for negative or positive control for immunoprecipitation. (**f**) Luciferase assay of P16 promoter activity with RRM1 mutant TDP-43 (F147/149L) overexpression. n = 8 replicates; one-way ANOVA with post hoc Tukey’s test. (**g**) Immunoblot analysis of FLAG-WT or 2 pathological TDP-43 mutants (G348C or A382T) overexpressed in HeLaS3 cells. Empty vector was transfected as the negative control. (**h-j**) Luciferase assay of P16 (**h**), P1 (upstream promoter) (**i**) and P2 (intron 1 promoter) (**j**) promoter activity with overexpression of WT TDP-43 or 2 pathological TDP-43 mutants (G348C or A382T). n = 7 (**h**), n = 3 (**i**) and n = 5 (**j**) replicates; one-way ANOVA with post hoc Tukey’s test. 2 μg of each reporter plasmids (P16, P1 and P2) were co-transfected with 1 μg of TDP-43/pFLAG-CMV2 in all overexpression experiments. Empty vector was used as the control in the overexpression experiment. All error bars indicate the means ± SEMs. *P < 0.05, **P < 0.01, ***P < 0.001, ****P < 0.0001.

Although the expression level of TDP-43 is strictly autoregulated via control of the stability of its own mRNA, whether TDP-43 acts on its own promoter as a transcriptional regulator is unclear. To determine the effects of TDP-43 on transcription, we conducted a promoter assay with repression or overexpression of TDP-43. We generated siRNA specifically targeting endogenous TDP-43 and confirmed the reduction in TDP-43 expression by immunoblotting (Fig. 5a). TDP-43 knockdown (KD) induced an 17.7% increase in the promoter activity of P16, while no effect in that of CMVp (Fig. 5b). In contrast, a significant reduction (16.6%) in promoter activity was observed with TDP-43 overexpression (Fig. 5c). Moreover, TDP-43 overexpression induced robust decrease of P16 activity (44.4%) under conditions of endogenous TDP-43 KD (Fig. 5d, Fig. S2b). Furthermore, chromatin immunoprecipitation (ChIP) assay revealed that either overexpressed FLAG-TDP-43 or endogenous TDP-43 binds to the *TARDBP* promoter (Fig. 5e). Previous study showed that RRM1 of TDP-43 is necessary and sufficient for DNA/RNA binding activity^26, 34^. Phe 147 and 149 located in the RRM1 form a canonical binding platform for nucleic acid association and mutations of these two key amino acids (F147/149L) is sufficient to abolish DNA/RNA binding and TDP-43 function^26, 37^. Our study demonstrated that TDP-43 (F147/149L) disrupted the negative effect for P16 activity (Fig. 5f). This result indicates that the DNA binding efficacy of TDP-43 via RRM1 is crucial for transcriptional autoregulation and the RRM1 mutant has lost the activity of transcriptional autoregulation. Similar to P16, TDP-43 overexpression significantly decreased P1 activity in a dose-dependent manner (Fig. 5i, Fig. S2d,e); however, it had no effect on P2 activity (Fig. 5j). These results indicate that TDP-43 has a role as a negative regulator of the *TARDBP* promoter in the transcriptional autoregulatory machinery circuit.

### Disruption of the autoregulatory machinery by TDP-43 mutants

To reveal whether disease-causing TDP-43 mutants affect the *TARDBP* promoter, we conducted a promoter assay with coexpression of the disease-causing mutants (G348C or A382T) (Fig. 5g–j). We confirmed that FLAG-TDP-43 proteins (WT, G348C and A382T) were expressed at the similar levels and localized in nuclei without aggregation (Fig. 5g, Fig. S2f). Furthermore, ChIP assay revealed that overexpressed pathological mutants (TDP-43 G348C and A382T) bound to the *TARDBP* promoter (Fig. S2g). While the WT TDP-43 exhibited a 22.4% reduction in P16 activity compared to that of the empty vector control, the G348C and A382T mutants did not affect promoter activity (Fig. 5h). We further demonstrated that the 2 pathological mutants also did not affect P1 (upstream promoter) activity in contrast to WT (Fig. 5i). In addition, the G348C and A382T mutants induced P2 (intron 1 promoter) activation significantly relative to that of the empty vector control or WT (Fig. 5j). Indeed, both the G348C and A382T mutants significantly induced P2 activity in a dose-dependent manner (Fig. S2h,i). These data indicate that TDP-43 with the G348C or A382T mutation disrupts the circuit of the transcriptional autoregulatory machinery.

### The *TARDBP* upstream region includes the predicted i-motif

**Figure 6:**
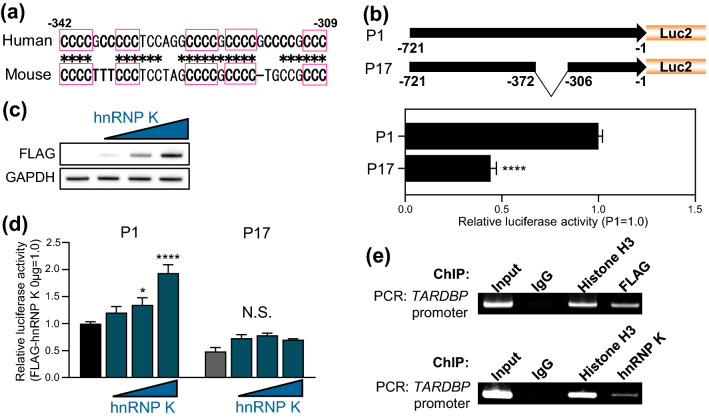
hnRNP K regulates the *TARDBP* promoter via the predicted i-motif region. (**a**) Conservation of C-rich sequences in predicted i-motif regions between humans and mice. The squares and asterisks indicate C-rich and conserved sequences, respectively. (**b**) Schematic representation and luciferase assay of the *TARDBP* promoter construct with deletion of the predicted i-motif (P17). n = 6 replicates; Welch’s t-test relative to P1. (**c**) Immunoblot analysis of FLAG-hnRNP K overexpression in HeLaS3 cells. (**d**) Luciferase assay of P1 and P17 promoter activity with dose-dependent hnRNP K overexpression. Each reporter plasmid was co-transfected with 0, 0.5, 1 and 2 μg of hnRNP K/pFLAG-CMV2. n = 3 replicates; two-way ANOVA with post hoc Dunnett’s test relative to 0 μg of hnRNP K/pFLAG-CMV2. All samples transfected 2 μg of reporter vector and 2 μg of pFLAG-CMV2 vector (empty vector + hnRNP K). All error bars indicate the means ± SEMs. *P < 0.05, ****P < 0.0001. (**e**) ChIP assay of overexpressed FLAG-hnRNP K (top) or endogenous hnRNP K (bottom) binding to the *TARDBP* promoter in HEK293T cells. Immunoprecipitation was performed using anti-FLAG antibody (for overexpressed FLAG-TDP-43) or anti-hnRNP K antibody (for endogenous hnRNP K), then PCR was conducted using primer detecting *TARDBP* − 721 to − 431 (290 bp). Control IgG and Histone H3 antibody are used for negative or positive control for immunoprecipitation.

Based on the study of deletion mutants of the upstream region (Fig. 2f,g), *cis*-acting elements important for regulatory factors are likely to be present in the region between nucleotides − 327 and − 223. We noted that this region includes C-rich sequences (− 327/− 309), which are highly conserved between humans and mice (Fig. 6a). A previous study showed that a C-rich strand can form i-motif DNA secondary structures^38–42^. Nuclease hypersensitivity element III_1_ (NHE III_1_) in the c-MYC promoter contains five runs of cytosines that can form multiple i-motif structures and strongly control the transcriptional activity of the c-MYC oncogene. Interestingly, the *TARDBP* sequence upstream of P1 includes nine runs of cytosines (− 371/− 309), a predicted i-motif region; thus, we investigated whether the predicted i-motif region is important for P1 activation (Fig. 1). P17 (Δ− 371/− 307), in which the predicted i-motif region is deleted, showed a 55.8% decrease in promoter activity relative to P1, suggesting that *cis*-acting elements exist in the − 371 to − 307 region (Fig. 6b). Heterogeneous ribonucleoprotein (hnRNP) K, a DNA/RNA-binding protein, is known to regulate the transcriptional activity of target genes by binding specifically to i-motifs^42–46^. Particularly, recent studies revealed the functional relationship between TDP-43 and hnRNP K in ALS^47, 48^. We thus hypothesized that hnRNP K may regulate *TARDBP* promoter activity as a part of the role of their co-regulation. Indeed, hnRNP K activated P1 and P16 in a dose-dependent manner but did not activate P17 or the CMVp (Fig. 6c,d, Fig. S3a,b). As overexpression of TDP-43 did not affect hnRNP K level, the negative effect of TDP-43 on *TARDBP* promoter was not due to the downregulation of hnRNP K (Fig. S3h). Furthermore, ChIP assay revealed that either overexpressed FLAG-hnRNP K or endogenous hnRNP K binds to the *TARDBP* promoter (Fig. 6e). We also conducted a promoter assay with repression of hnRNP K and remarkable reduction in P1 activity (68.4%) was observed with hnRNP K KD (Fig. S3c,d). These data suggest that *TARDBP* promoter activity is regulated by hnRNP K via the region (− 371 to − 307) which includes predicted i-motif. Since insufficiency of hnRNP K affect the activity of CMVp (38.2%), it is suggested that hnRNP K may also have a role in the general promoter (Fig. S3e).

## Discussion

In the present study, we experimentally identified the promoter region of the *TARDBP* gene for the first time. A previous study predicted the *TARDBP* promoter region using two prediction software packages, ElDorado and Promoter Scan^36^. *TARDBP* promoter regions were predicted in the 502 nucleotides upstream of exon 1, across exon 1 and 66 nucleotides downstream of exon 1 and in two regions of intron 1 (212 and 613 nucleotides) by ElDorado and in the − 577 to − 326 region by Promoter Scan. Our promoter prediction via the UCSC genome browser also indicated the existence of promoters in the *TARDBP* upstream region and intron 1 (Fig. 2a). Consistent with the predictions, marked promoter activity was experimentally detected in the upstream − 721/− 1 region, and intron 1 also exhibited relatively weak promoter activity—approximately 25% that of the upstream promoter—in 3 cell lines (Fig. 2c–e). However, it is not clear whether the intron 1 promoter is active and can start transcription from exon 2 in vivo. Then, we attempted to search a database (DataBase of Transcriptional Start Sites (DBTSS), https://dbtss.hgc.jp) that presents the exact positions of TSSs in the genome^49^. We found that transcription actually starts from TSS2 in most TDP-43 transcripts, but only a minor population of TDP-43 transcripts in the human fetal brain have a TSS within exon 2 (data not shown). We also showed that the proximal half of *TARDBP* intron 1, the region that includes a critical element for promoter activity, is highly conserved in chimpanzees and macaques but not in mice, dogs, chickens or zebrafish (Fig. 3d). This finding indicates that the intron 1 regulatory element may have been acquired by primates via evolution.

Generally, it is known that splicing is a post-transcriptional event enhancing the gene expression. Indeed, the sequential region of *TARDBP* (upstream/exon 1/intron 1), whose intron 1 was spliced out producing the transcript with exon 1 (Fig. S2a), exhibited 2.60-fold stronger activity than the upstream promoter alone (Fig. 4b). Interestingly, P16 (Δintron 1), which produces same transcript as P16 without splicing, exhibited 2.58-fold increase the promoter activity than P16 (Fig. 4c). There is no evidence supporting that exon 1 has an influence on the upstream promoter activity, however it may enhance the gene expression by posttranscriptional mechanisms as a 5′UTR of the transcript.

In present study, we demonstrated a regulatory effect of hnRNP K on *TARDBP* upstream promoter activity. hnRNP K has multiple regulatory functions in events such as chromatin remodeling, transcription, splicing, mRNA stability and translation processes^45^. Regarding transcriptional regulation, hnRNP K activates the c-src promoter and promotes its transcription in cooperation with the transcription factor Sp1^45, 46^. In contrast, hnRNP K has suppressive effects on several transcriptional targets. Indeed, hnRNP K suppresses the thymidine kinase gene promoter and inhibits Sp1- and Sp3-mediated transactivation of the neuronal nicotinic acetylcholine receptor β4 subunit^45, 50^. Previous reports demonstrated that hnRNP K regulates the transcription of its target promoter by interacting with the i-motif, a unique secondary structure of single-stranded DNA. The i-motif is a tetraplex structure formed by hemiprotonated cytosine-cytosine (C–C^+^) base pairing under acidic conditions in DNA sequences enriched in cytosine^38–42^. In fact, hnRNP K is reported to bind selectively to the i-motif and positively modulate KRAS transcription^40, 44^. In our study, ChIP assay experiment revealed the interaction between hnRNP K and *TARDBP* promoter region (Fig. 6e) while it is still unclear whether hnRNP K directly binds to the predicted i-motif of the *TARDBP* upstream promoter. Since the P17 reporter construct lacking the predicted i-motif region showed a reduction in promoter activity to half of that of P1 (Fig. 6b) and lost responsiveness to hnRNP K (Fig. 6d), indicating that this region is a critical *cis*-acting element for *TARDBP* upstream promoter activity and for transcriptional activation by hnRNP K. In fact, the predicted i-motif region of the *TARDBP* upstream promoter is highly conserved between humans and mice (Fig. 6a), which supports our conclusion. Unfortunately, we could not show the regulatory effect of hnRNP K on the transcription of the endogenous *TARDBP* (Fig. S3f,g), however it might be due to the potent autoregulation system of TDP-43 which makes it difficult to prove. Recent reports demonstrated the functional interaction between hnRNP K and TDP-43. hnRNP K has been shown to bind to TDP-43 in the cytoplasm and facilitate the control of TDP-43 trafficking. Moreover, attenuated expression of hnRNP K is observed in ALS patient fibroblasts and in mouse ALS models^47^. Indeed, there is a global reduction of hnRNP K expression in ALS spinal cord motor neurons compared to healthy controls^48^. Thus, the accumulating evidence strongly suggests a potential association of hnRNP K with ALS pathogenesis via TDP-43 regulation. The present study indicates a role for hnRNP K in controlling TDP-43 which led us to further explore the physiological and pathophysiological significance of the mutual regulation between TDP-43 and hnRNP K.

C9orf72 is the most common causative gene of both fALS and sALS, and GGGGCC repeat expansion has been reported in the UTR of C9orf72^3, 15, 16^. A GGGGCC hexanucleotide repeat expansion is located in the first intron of the C9orf72 gene, and ALS patients possess 500 to > 5000 repeats, whereas individuals without disease possess 2–19 repeats^15, 16^. A recent study reported that the antisense GGCCCC repeat forms an i-motif and that hnRNP K preferentially binds to the antisense repeat^51–53^. This finding implies that the excessive presence of i-motifs in ALS patients with C9orf72 repeat expansion may trap i-motif-binding proteins, such as hnRNP K, and affect the *TARDBP* promoter.

Our results suggest that the transcriptional regulation of TDP-43 by its own promoter is also involved in the autoregulatory machinery in a manner controlled by mRNA stability. TDP-43 KD significantly increased P16 activity (Fig. 5b), indicating that TDP-43 has a negative effect on its own promoter activity. Indeed, overexpression of TDP-43 significantly reduced the activity of P16 and P1 upstream promoter to 83.4% and 73.9% that of baseline, respectively (Fig. 5c,i). These findings indicate that TDP-43 is a negative regulator of the upstream promoter. Our ChIP assay experiment revealed that TDP-43 binds to the *TARDBP* promoter region (Fig. 5e), therefore our findings imply that the transcriptional regulation of *TARDBP* promoters by TDP-43 plays an important role in the autoregulation of the TDP-43 level. In conclusion, the TDP-43 expression is strictly regulated to an appropriate level by multiple autoregulatory mechanisms.

As reported previously, either an excess or deficiency of TDP-43 induced MN disorders^29–32^; thus, elucidating the involvement of mutant TDP-43 in TDP-43 autoregulation is very important. Regarding the 2 most common mutants of TDP-43^54^ used in the present experiments, G348C and A382T, 2 mutants tend to induce comparatively early onset of fALS^55^. Yamanaka and colleagues reported that the mutants exhibited longer half-lives than WT TDP-43 and speculated that the longer half-lives of these mutant proteins were correlated with accelerated ALS onset^55^; G348C and A382T mutants belong to the longest half-life group among 7 disease causing mutants (G298S, A315T, M337V, Q343R, G348C, N352S and A382T). Our transient expression studies demonstrated that there are no significant differences in the subcellular localization pattern and the promoter binding ability among WT and mutant TDP-43s (Fig. S2f,g).

In particular, among the 2 mutants selected in our study, the A382T mutant exhibited the longest half-life and was associated with the earliest ALS onset, indicating that A382T is one of the most “malignant” mutants. In our experiment, compared with WT TDP-43, A382T did not affect *TARDBP* upstream promoter activity but induced significant activation of the intron 1 promoter (Fig. 5i,j). In other words, A382T is not involved in the negative feedback loop via the *TARDBP* upstream promoter and can maintain a high level of expression by escaping from the autoregulatory machinery.

Our investigation succeeded in experimentally detecting promoter activity in the *TARDBP* upstream region. As WT TDP-43 but not mutant TDP-43 negatively regulated its own promoter activity, our findings suggest that the TDP-43 mutants may contribute to maintaining high levels of TDP-43 transcription and promoting the early onset and progression of ALS.

## Methods

### DNA construction and siRNA

Human *TARDBP* upstream and intronic genomic DNA were isolated from human induced pluripotent stem cell (hiPSC)-derived neural stem cells (201B7) using TRIzol reagent (Invitrogen) following the manufacturer’s instructions. The cloned genome and luciferase (Luc2) gene liberated from the Luc2/pGL4 vector (Promega) were subcloned into the pcDNA3.1 (−) vector (Invitrogen). Promoter fragment/Luc2 constructs were produced using the primers described in Table S3. The hnRNP K/pFLAG-CMV2 plasmid used for hnRNP K overexpression was a gift from Dr. Yano. For TDP-43-Venus overexpression, TDP-43-Venus/pcDNA3 and *TARDBP* upstream region-TDP-43-Venus/pcDNA3.1(−) were constructed using the former plasmid. For the promoter assay, TDP-43 (WT, G348C, A382T and F147/149L) were cloned into the pFLAG-CMV2 vector (Sigma). All primer sequences used for plasmid construction are described in the Supplemental Information (Tables S2, S3). PCR for the construction was conducted using PrimeSTAR MAX (Takara Bio) according to the manufacturer’s protocol.

For TDP-43 KD, double-stranded siRNA targeting TDP-43 was originally designed using siDirect software (Version 2.0, http://sidirect2.rnai.jp/). The target and negative control siRNA sequences are described in Table S1.

### Cell culture

HEK293T and HeLaS3 cells were cultured in DMEM (Sigma) containing 10% FBS (GE Healthcare), nonessential amino acids (NEAA) (Gibco) and 1% penicillin–streptomycin (Wako). SH-SY5Y cells were maintained in DMEM/F12 (Sigma) containing 10% FBS, 1% penicillin–streptomycin and NEAA. hiPSCs (201B7) were purchased from RIKEN Cell Bank and maintained in StemFit medium (Ajinomoto). Neurospheres were produced and maintained according to a previous report^56^. Briefly, cells were cultured in KBM neural stem cell medium (Kohjin Bio) supplemented with 2% B27 (Gibco), 20 ng/mL bFGF (Wako), 10 ng/mL LIF (Millipore), 10 ng/mL Y-27632 (Wako), 3 μM CHIR (Cayman Chemical Company), 2 μM SB431542 (Sigma), 1 μM retinoic acid (Wako), and 1 μM purmorphamine (Cayman Chemical Company).

### Luciferase assay

For plasmid transfection, cells were transfected with 2 μg of promoter/luciferase vector (or 1 μg of CMV promoter/luciferase vector) using Lipofectamine 3000 Reagent (Invitrogen) according to the manufacturer’s instructions. After transfection for 24 (HeLaS3 and HEK293T) or 48 h (SH-SY5Y), a promoter assay was performed using a luciferase assay system (Promega) according to the manufacturer’s instructions. Briefly, cells were collected with the extraction buffer provided in the kit and centrifuged at 12,000×*g* for 2 min at 4 °C. The supernatants were collected for the conduction of subsequent experiments. Luminescence detection was performed using a WSE-6100H LuminoGraph I (Atto). The signal quantification was conducted ImageJ (Fiji) software (Version 1.53, National Institute of Health)^57^.

For multiple plasmid cotransfection, HeLaS3 cells were transfected with 2 μg of promoter/luciferase vector; 0, 0.5, 1 or 2 μg of FLAG-hnRNP K or TDP-43 plasmid; and 2, 1.5, 1 or 0 μg of pFLAG-CMV2 empty vector to equal total 4 μg of plasmid DNA in each group. For siRNA and plasmid DNA cotransfection, HeLaS3 cells were transfected with 60 nM (final conc.) siRNA using Lipofectamine RNAiMAX (Invitrogen), and after 24 h, additional transfection was performed using Lipofectamine 3000 with 2 μg of promoter/luciferase vector. After another 24 h, a luciferase assay was conducted.

### Immunocytochemistry and fluorescence observations

For immunocytochemistry, HeLaS3 cells transfected with the 1 μg of TDP-43 (WT, G348C or A382T) /pFLAG-CMV2 fixed with 4% paraformaldehyde (PFA) for 5 min at room temperature (RT) and washed with PBS after 24 h of transfection, and permeabilized with 0.01% Triton X-100 for 15 min at RT. After washing with PBS and incubation in 5% BSA for one hour at RT, cells were incubated with the primary antibodies, anti-DDDDK-tag (MBL M185-3 1:1000), for overnight at 4 °C. The cells were washed with PBS, then incubated with the secondary antibody Alexa 488-conjugated antibody (Molecular Probes A11001, 1:200) and Hoechst 33342 (Molecular Probes H3570, 1:1000), for one hour at RT. After washing with PBS, fluorescence was observed using a confocal laser scanning microscope (Zeiss) with a Plan Apochromat 20×/0.8 objective. For promoter reporter overexpression and nuclear staining, HeLaS3 cells transfected with 1 μg of the Venus reporter vector (driven by CMV promoter or P1) were fixed with 4% PFA for 5 min RT and washed with PBS after 48 h of transfection. Incubation with Hoechst 33342 was performed for one hour. After washing with PBS, fluorescence was visualized using the confocal microscope with a Plan Apochromat 63×/1.4 oil immersion objective. Comparison images were acquired with the same laser intensity, exposure time, and filter. Details are described in each figure legend.

### RT-PCR analysis

Total RNA from HeLaS3 cells transfected P16 or P16 (Δintron 1) was extracted using TRIzol reagent and RNeasy Plus Mini Kit (Qiagen). cDNA synthesis and PCR were performed using ReverTra Ace α (TOYOBO) and EmeraldAmp PCR Master Mix (TaKaRa) following the manufacturer’s instructions. Primers for the detection of the transcript of exon 1/intron 1/luciferase are described in Table S4.

### Immunoblotting

Cultured cells were washed with PBS, and protein was extracted with extraction buffer containing Tissue Extraction Reagent I (Wako), protease inhibitor cocktail (Roche) and a phosphatase inhibitor (Nacalai Tesque) on ice for 10 min. The extracted solutions were transferred into 1.5 mL tubes and centrifuged at 15,000 rpm for 15 min at 4 °C. Supernatants were collected in new tubes and used for subsequent experiments. Protein lysates were mixed with 2 × Laemmli sample buffer (Bio-Rad) and 2-mercaptoethanol (Sigma) and boiled for 3 min at 95 °C. Protein samples were separated by electrophoresis on 10% TGX FastCast Acrylamide gels (Bio Rad) and transferred to Immobilon-P membranes (Millipore). Nonspecific antibody reactions were blocked with 1% skim milk (Wako) for 30 min at RT. Membranes were incubated with primary antibody at 4 °C overnight. Membranes were then washed with TBST and incubated with horseradish peroxidase (HRP)-conjugated secondary antibodies for 2 h at RT. After a wash in TBST, protein signals were detected by Western BLoT Chemiluminescence HRP Substrate (Takara Bio) using the WSE-6100H LuminoGraph I (Atto). The following primary antibodies were used: anti-TDP-43 (Protein Tech 10782-2-AP, 1:1000), anti-hnRNP K (MLB RN019P, 1:1000), anti-DDDDK (FLAG)-tag (MBL M185-3, 1:10000), anti-GAPDH (Cell Signaling #5174, 1:1000), and anti-β-actin (Sigma A1978, 1:2000). The secondary antibodies are described below: HRP-conjugated goat anti-rabbit IgG (Millipore AP307P, 1:2000) and HRP-conjugated goat ant-mouse IgG (Millipore AP308P, 1:2000).

### Chromatin immunoprecipitation (ChIP) assays

ChIP assays were performed using SimpleChIP Enzymatic Chromatin IP Kit (Cell Signaling) as described by manufacturer’s protocol. Briefly, HEK293T cells were fixed in 1% formaldehyde for 10 min at RT, quenched, and enzymatically digested for 20 min at 37 °C. For overexpression analysis, the cell fixation was performed after 24 hr from transfection. Chromatin was briefly sheared by sonication (three times, 20 s each), and was incubated overnight at 4 °C with specific antibody, anti-DDDDK (FLAG)-tag (MBL M185-3, 6.7 ng/μL), anti-TDP-43 (Protein Tech 10782-2-AP, 1.0 ng/μL) or anti-hnRNP K (MBL RN019P, 10 ng/μL). Control IgG and Histone H3 antibody were provided with the ChIP kit as a negative or positive control for the immunoprecipitation. Approximately 4 × 10^6^ cells were used for all immunoprecipitations. Immunoprecipitated chromatin was then incubated with protein G magnetic beads, washed and eluted. After reversal of the cross-links and purification, precipitated DNAs were analyzed by PCR with specific primers using EmeraldAmp PCR Master Mix (TaKaRa). The primers are described in Table S5.

### Statistical analysis and graphs

Statistical data were analyzed by GraphPad Prism 7.03 software (GraphPad Software). The significance level was set at 0.05, and Welch’s t test (1 variable, 2 groups), one-way ANOVA (1 variable, > 2 groups) and two-way ANOVA (2 variable, > 2 groups) were used. After ANOVA, additional analysis was performed utilizing Tukey’s or Dunnett’s post hoc test, and details are provided in the figure legends.

## Supplementary Information

Supplementary Information 1.
