# Supplementary material for: Characterization of the upstream and intron promoters of the gene encoding TAR DNA-binding protein
Source: Sci Rep. 2021 Apr 22;11:8720. doi: 10.1038/s41598-021-88015-y (PMC8062691; doi:10.1038/s41598-021-88015-y)
Supplement: Supplementary file 1 — Supplementary Information 1. [file 41598_2021_88015_MOESM1_ESM.pdf]

## Supplemental information

# Characterization of the upstream and intron promoters of the gene encoding TAR DNA-binding protein.

Minami Hasegawa-Ogawa[1], Hirotaka James Okano[1*]
1. Division of Regenerative Medicine, The Jikei University School of Medicine, 3-25-8 Nishi-Shimbashi, Minato-ku, Tokyo 1058461, Japan

**Figure S1**

```
+1     GGTGGGCGGG GGGAGGAGGC GGCCCTAGCG CCATTTTGTG GGAGCGAAGC GGTGGCTGGG CTGCGCTTGG GTCCGTCGCT
       ↑                                        Exon1
       TSS2
+81    GCTTCGGTGT CCCTGTCGGG CTTCCCAGCA GCGGCCTAGC GGGTGAGTCG CGGAGCCTTC TCACTGAGGA CGCCGTAGGT
                                                      1 Fwd

+161   GCCTCGGCTT CTGCATTGGG GCGGCCCGAG GCAGACGCAG CGGCTTCGGA CCCTGCCCCG GGAAGTCAGC CGTGAGACCG

+241   GGCCTAGTCC TGCCCGAGCG GGCTCGCGCG GGAGCGTCGT TAGGTCCTGG CACGGGGAGG CCGGTGGCGC CGAAACGGGG

+321   GGCCGGGCCC GCGGTGCCGC GTAGCCTGAG GCGGAGGTTG GCCCCGGACC CGGACAGTGC CGGAGCCTTC GGTACTTTGT

+401   ACTTCGAGGG ACGCTGCCAG CTTGGGCCTG GTGCTCCTGC GCCCCACCCC GGGCCGCGCC GAGAAACTCC TCCCTCGGCT

+481   GGCACGCGCG GGCTTCTGGG AGGCGACGGC CGGCACCGCC AGGACCTGTC GCCGCGGGCC TTGGGGCTCG GGCTCGCAGA

+561   GCCCCCGGAA GCCCCAAAAT GAGGAACAGA GGGAAACTTT TCTCCTGTTC TTTACACCTG GTTCACTGCC CATATTCAGT
                                                           500 Rev           501 Fwd

+641   CTTCAGGTTC AGCTGATGGA TCGCTTGCTT AGGAAAATCG ACTGGGACCT ATCACGCCCA TGCCTCAGCC AGTTAGGCTC

+721   CTCCTCTGCC TTCAGGATTA CTTAACGACT AACCCGATTG TCATAAAATA GCTACTGTTT ATTGTTCCCT TGCATACCCT
           603 Fwd                                                            666 Fwd

+801   AGCTTGTAAC CAGCAAGCCA CGTTGGACTC ACAGTTACAG TTTCAGTACC TGACGCATCA TAAGCCTTCA GGGAAAGTTT
                                                                          737 Fwd

+881   TTTGAATGAA TGAACTGGCG AGGCATCACA TTTTGATAGG AAATCACTAC CCTTACCTTC ACCTCGTCAT TTTTCAGGGA

+961   TAACCAATGC ATATACGAAT CCAGACAAGC ATTTTTCTGG AAGTCAGAAC TCTGACATGG TTTGGGTATT ATCATTATAA
                      850 Fwd

+1041  GGAAACAGTT ATTCTGACAT GAATGTTGTT CATTCATATC TCTTTTCTCT TTAG +1094
                                              972 Rev
```

**Figure S1. *TARDBP* exon 1 and intron 1 sequences and primers.** *TARDBP* noncoding exon 1 (+1 to +122), and all 972 nucleotides of *TARDBP* intron 1, (+123 to +1094), with respect to TSS2. The highlighted sequences indicate exon 1. Underlining denotes primer sequences. Fwd; forward primer, Rev; reverse primer.

Figure S2

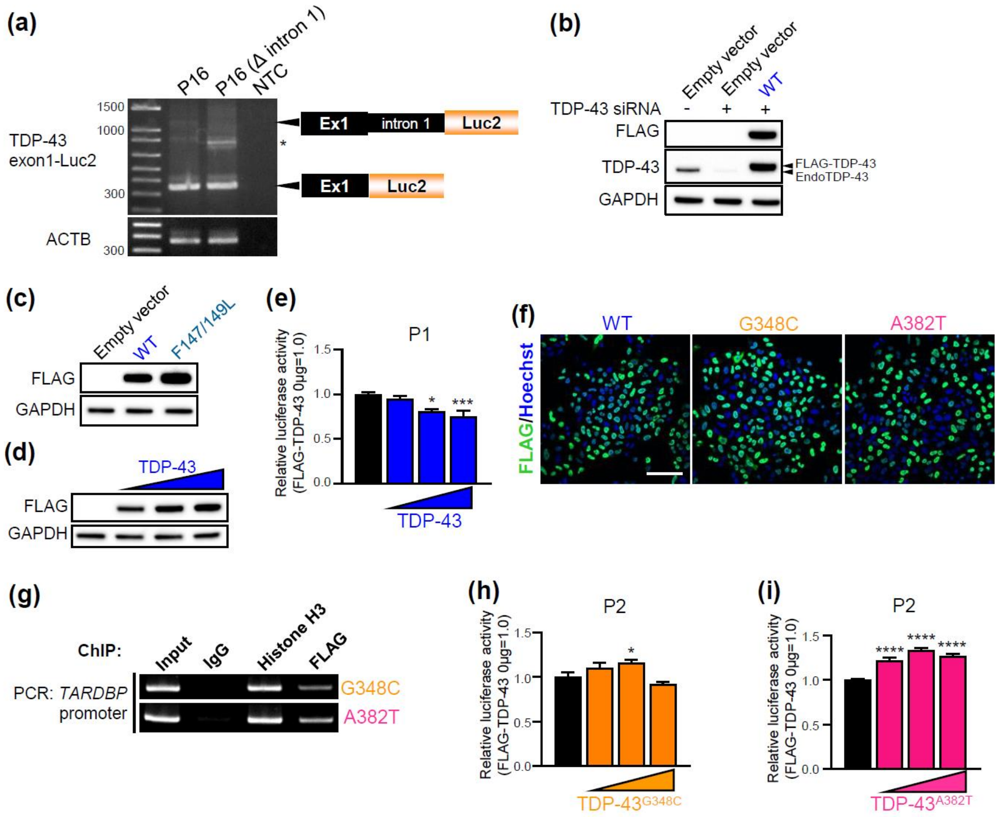


**Figure S2. TDP-43 affects the activity of the *TARDBP* upstream and intron 1 promoters.** (**a**) RT-PCR analysis of *TARDBP* exon1 to Luc2 and actin-beta mRNA expression in HeLaS3 cells overexpressing P16 or P16 (Δintron 1). Forward and reverse primers are set at *TARDBP* exon1 and Luc2 to detect intron 1 splicing. Amplicon length are following; non-spliced product (exon1-intron 1-Luc2); 1294bp and spliced product (exon1-Luc2); 322bp, respectively. Asterisk indicates non-specific band. (**b-d**) Immunoblot analysis of endogenous TDP-43 KD and exogenous FLAG-WT TDP-43 overexpression (**b**), FLAG-WT TDP-43 or mutant TDP-43 (F147/149L) overexpression (**c**) and FLAG-WT TDP-43 dose-dependent overexpression (**d**) in HeLaS3 cells. Mock siRNA and/or Empty vector was transfected as the negative control. (**e**) Luciferase assay of P1 (upstream promoter) in HeLaS3 cells overexpressing dose-dependent FLAG-WT TDP-43. n=9 replicates; one-way ANOVA with *post hoc* Dunnett's test relative to 0μg of TDP-43/pFLAG-CMV2. (**f**) Immunocytochemistry images of FLAG-TDP-43 (WT, G348C or A382T) overexpressing HeLaS3 cells after 24hr from transfection. Nuclei were stained with Hoechst (blue). Scale bar: 100 μm. (**g**) ChIP assay of overexpressed 2 pathological mutant TDP-43 (G348C or A382T) binding to the *TARDBP* promoter in HEK293T cells. Immunoprecipitation was performed using anti-DDDDK (FLAG) antibody, then PCR was conducted using primer detecting

*TARDBP* -721 to -431 (290bp). Control IgG and Histone H3 antibody are used for negative or positive control for immunoprecipitation. (**h, i**) Luciferase assay of P2 (intron 1 promoter) in HeLaS3 cells overexpressing dose-dependent FLAG-TDP-43 (G348C) (**h**) and FLAG-TDP-43 (A382T) (**i**). n=6 replicates; one-way ANOVA with *post hoc* Dunnett's test relative to 0μg of TDP-43/pFLAG-CMV2. All samples transfected 2 μg of reporter vector and 2 μg of pFLAG-CMV2 vector (empty vector + TDP-43). All error bars indicate the means ± SEMs. *$P$<0.05, ***$P$<0.001, **** $P$<0.0001.

Figure S3

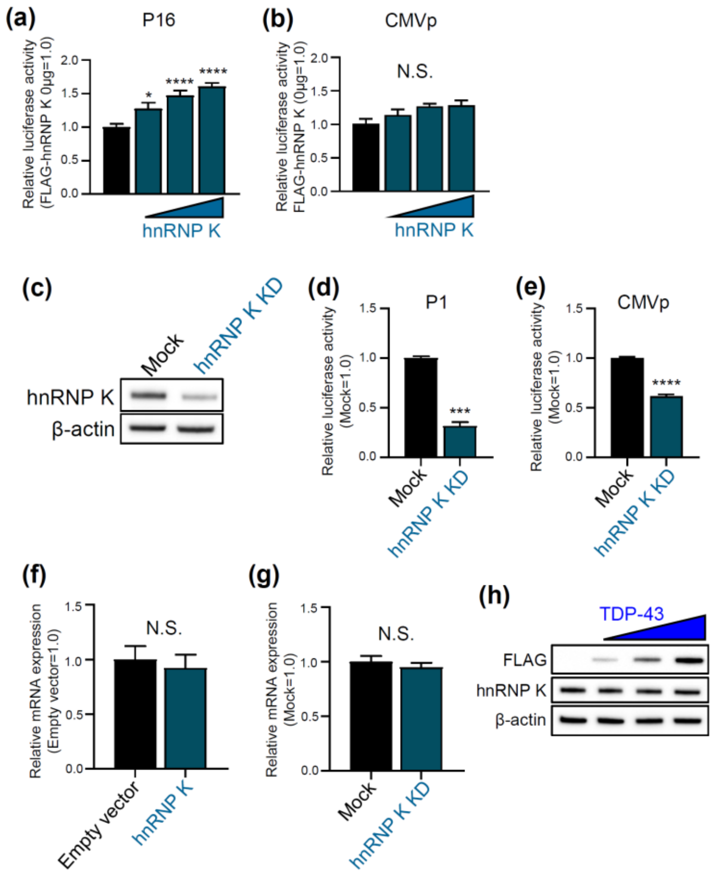


**Figure S3. hnRNP K does not affect intron 1 or CMV promoter activity.** (**a, b**) Dose-dependent effects of hnRNP K on P16 (**a**) or the CMV promoter (**b**). n=9 (**a**), n=3 (**b**) replicates, one-way ANOVA with *post hoc* Dunnett's test relative to 0μg of hnRNP K/pFLAG-CMV2. All samples transfected 2 μg of reporter vector and 2 μg of pFLAG-CMV2 vector (empty vector + hnRNP K). (**c**) Immunoblot analysis of hnRNP K in HeLaS3 cells. Mock and KD indicate transfected negative control or hnRNP K siRNA, respectively. (**d, e**) Luciferase assay of P1 (**d**) or CMV promoter (**e**) activity with hnRNP K KD. n=3 (P1) (**d**), n=4 (CMVp) (**e**) replicates; Welch's t-test relative to Mock. (**f, g**) mRNA expression of endogenous TDP-43 with FLAG-hnRNP K overexpression (**f**) or hnRNP K KD (**g**). n=3 replicates, Welch's t-test relative to Empty vector (**f**) or Mock (**g**). (**h**) Immunoblot analysis of hnRNP K with dose-dependent overexpression of FLAG-WT TDP-43. All error bars indicate the means ± SEMs. *$P$<0.05, ***$P$<0.001, **** $P$<0.0001.

**Figure S4**

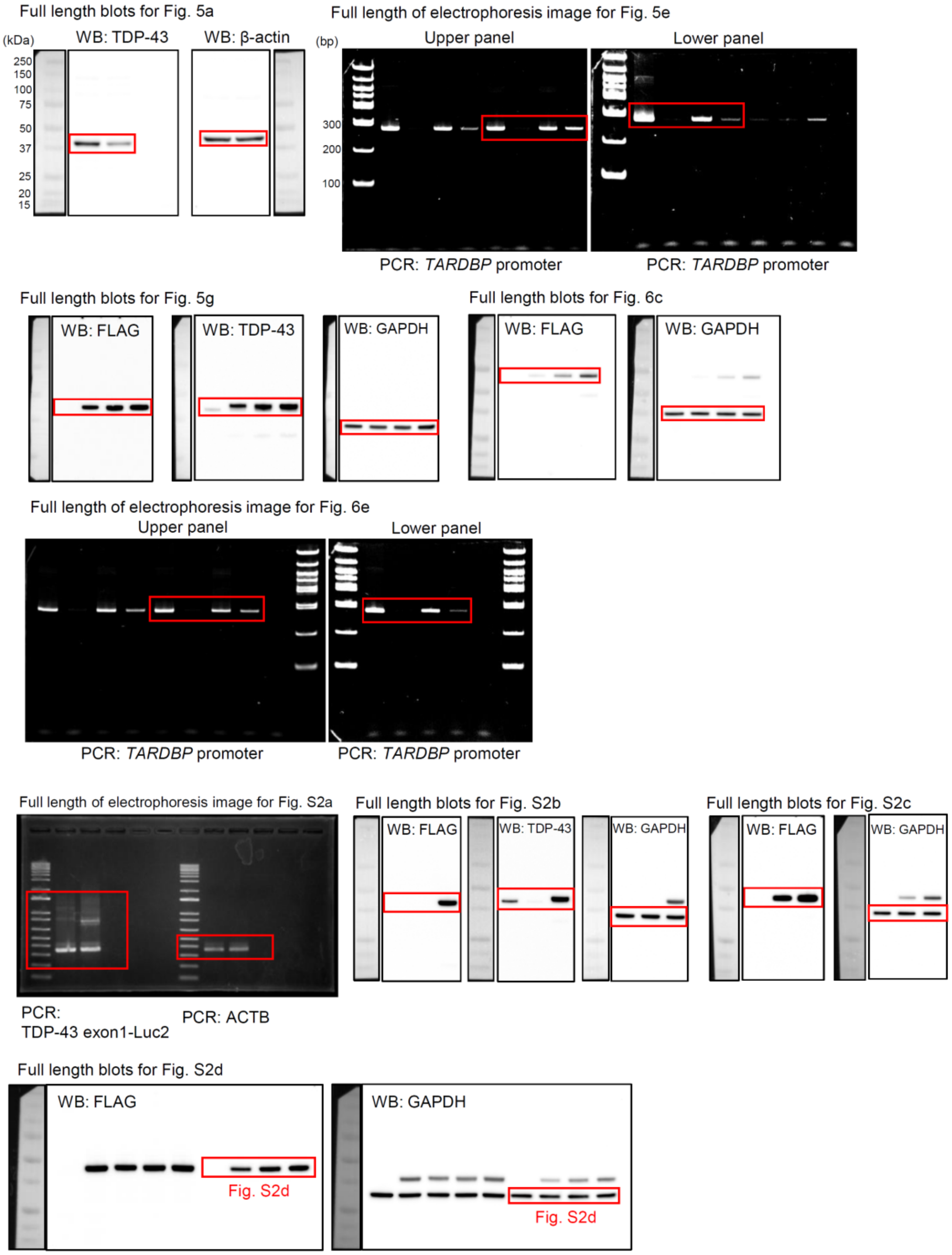
Full length blots for Fig. 5a
(kDa)
WB: TDP-43
WB: β-actin
250
150
100
75
50
37
25
20
15
Full length of electrophoresis image for Fig. 5e
(bp)
Upper panel
Lower panel
300
200
100
PCR: TARDBP promoter
PCR: TARDBP promoter
Full length blots for Fig. 5g
WB: FLAG
WB: TDP-43
WB: GAPDH
Full length blots for Fig. 6c
WB: FLAG
WB: GAPDH
Full length of electrophoresis image for Fig. 6e
Upper panel
Lower panel
PCR: TARDBP promoter
PCR: TARDBP promoter
Full length of electrophoresis image for Fig. S2a
PCR: TDP-43 exon1-Luc2
PCR: ACTB
Full length blots for Fig. S2b
WB: FLAG
WB: TDP-43
WB: GAPDH
Full length blots for Fig. S2c
WB: FLAG
WB: GAPDH
Full length blots for Fig. S2d
WB: FLAG
Fig. S2d
WB: GAPDH
Fig. S2d

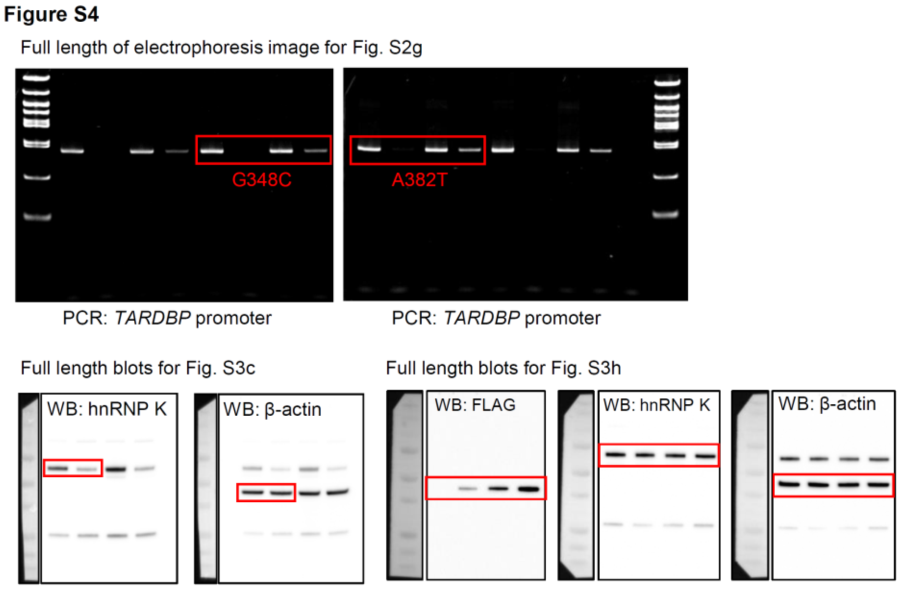


**Figure S4. Full length blots or PCR images used to generate main and supplemental figures.**

**Table S1. siRNA sequences for TDP-43 KD experiments.**

| siRNA | siRNA Sequence |
|---|---|
| TDP-43 siRNA passenger | 5′-GGUUUCUCCUGUAAUAUUUUA-3′ |
| TDP-43 siRNA guide | 5′-AAAUAUUACAGGAGAAACCUU-3′ |
| Negative control siRNA passenger | 5′-GUAAACGGCCACAAGUUCAGC-3′ |
| Negative control siRNA guide | 5′-UGAACUUGUGGCCGUUUACGU-3′ |

**Table S2. Primers used for construction of TDP-43 tagged with N-terminal FLAG.**

| For production of TDP-43 tagged with N-terminal FLAG | |
|---|---|
| **Gene** | **Primer Sequence** |
| FLAG-TDP-43 (WT) | Forward: 5′-CAAGCTTTCTGAATATATTCGGGTAAC-3′<br>Reverse: 5′-GGGTACCTTACATTCCCCAGCCAGAAGAC-3′ |
| FLAG-TDP-43 (F147/149L) | Forward: 5′-AGGGGCTGGGCCTGGTTCGTTTTACGGAATATG-3′<br>Reverse: 5′-GAACCAGGCCCAGCCCCTTTGAATGACCAGTCT-3′ |
| FLAG-TDP-43 (G348C) | Same with TDP-43 (WT) |
| FLAG-TDP-43 (A382T) | Same with TDP-43 (WT) |
| **For production of TDP-43 tagged with C-terminal Venus driven by CMV** | |
| TDP-43 | Forward: 5′- TGGGCCCCCACCATGTCTGAATATATTCGGGTAAC-3′<br>Reverse: 5′-GGGTACCCATTCCCCAGCCAGAAGAC-3′ |
| Venus | Forward: 5′-CGGATCCGTGAGCAAGGGCGAGG-3′<br>Reverse: 5′- CGGATCCGTGAGCAAGGGCGAGG -3′ |
| **For production of TDP-43 tagged with C-terminal Venus driven by P1** | |
| TDP-43 | Forward: 5′-TTGCGGCCGCCCACCATGTCTGAATATATTCGG-3′<br>Reverse: 5′-GGGTACCCATTCCCCAGCCAGAAGAC-3′ |
| Venus | Forward: 5′-CGGATCCGTGAGCAAGGGCGAGG-3′<br>Reverse: 5′-CAAGCTTTTACTTGTACAGCTCGTCCATG-3′ |

**Table S3. Primers used for construction of *TARDBP* upstream and intron fragments fused with Luc2.**

| Cloning *TARDBP* upstream -721/-1 | |
|---|---|
| **721/-412** | Forward: 5′-GTCAATTGCGCCTACCGCGTTCAAGCAATTC-3′<br>Reverse: 5′-TAGTGCGTCTTCTCCTCCCT-3′ |
| **-431/-281** | Forward: 5′-AGGGAGGAGAAGACGCACTA-3′<br>Reverse: 5′-GAGAGTAGCACAGAGTCTCG-3′ |
| **-300/-1** | Forward: 5′-CGAGACTCTGTGCTACTCTC-3′<br>Reverse: 5′-TTGCGGCCGCGGCGTCCTCTCCCACCGGTTG-3′ |
| ***TARDBP* upstream promoter region** | |
| **Name** | **Primer Sequence** |
| **P1<br>(-721/-1)** | Forward: 5′-GTCAATTGCGCCTACCGCGTTCAAGCAATTC-3′<br>Reverse: 5′-TTGCGGCCGCGGCGTCCTCTCCCACCGGTTG-3′ |
| **P4<br>(-300/-1)** | Forward: 5′-GTCAATTGCGAGACTCTGTGCTACTCTC-3′<br>Reverse: 5′-TTGCGGCCGCGGCGTCCTCTCCCACCGGTTG-3′ |
| **P5<br>(-721/-223)** | Forward: 5′-GTCAATTGCGCCTACCGCGTTCAAGCAATTC-3′<br>Reverse: 5′-TTGCGGCCGCCGGCCGACCAACCGCCGGCC-3′ |
| **P6<br>(-721/-281)** | Forward: 5′-GTCAATTGCGCCTACCGCGTTCAAGCAATTC-3′<br>Reverse: 5′-TTGCGGCCGCGAGAGTAGCACAGAGTCTCG-3′ |
| **P7<br>(-527/-223)** | Forward: 5′-GTCAATTGTGTGAATACTCAGAAAGTACCTGGC-3′<br>Reverse: 5′-TTGCGGCCGCCGGCCGACCAACCGCCGGCC-3′ |
| **P8<br>(-527/-281)** | Forward: 5′-GTCAATTGTGTGAATACTCAGAAAGTACCTGGC-3′<br>Reverse: 5′-TTGCGGCCGCGAGAGTAGCACAGAGTCTCG-3′ |
| **P9<br>(-527/-327)** | Forward: 5′-GTCAATTGTGTGAATACTCAGAAAGTACCTGGC-3′<br>Reverse: 5′-TTGCGGCCGCCCTGGAGGGGGCGGGGCTCGGGATC-3′ |
| **P17<br>(*del* -371/-307)** | Forward1: 5′-GTCAATTGCGCCTACCGCGTTCAAGCAATTC-3′<br>Reverse1: 5′-GCTCGAGGCAGGATAAGGCCTGGAC-3′<br>Forward2: 5′-CCTCGAGCTGGCCGAGACTCTGTGC-3′<br>Reverse2: 5′-TTGCGGCCGCGGCGTCCTCTCCCACCGGTTG-3′ |
| ***TARDBP* intron1 (+123/+1094, total 972 nucleotides)** | |
| **P2<br>(1/972)** | Forward: 5′-GCCAATTGGTGAGTCGCGGAGCCTTC-3′<br>Reverse: 5′-TTGCGGCCGCCTAAAGAGAAAAGAGATATG-3′ |
| **P10<br>(1/500)** | Forward: 5′-GCCAATTGGTGAGTCGCGGAGCCTTC-3′<br>Reverse: 5′-TTGCGGCCGCACCAGGTGTAAAGAACAG-3′ |
| **P11<br>(501/972)** | Forward: 5′-GTCAATTGTCACTGCCCATATTCAG-3′<br>Reverse: 5′-TTGCGGCCGCCTAAAGAGAAAAGAGATATG-3′ |

| | |
|---|---|
| **P12 (603/972)** | Forward: 5′-GCCAATTGTCTGCCTTCAGGATTAC-3′<br>Reverse: 5′-TTGCGGCCGCCTAAAGAGAAAAGAGATATG-3′ |
| **P13 (666/972)** | Forward: 5′-GCCAATTGCCTTGCATACCCTAGCTTG-3′<br>Reverse: 5′-TTGCGGCCGCCTAAAGAGAAAAGAGATATG-3′ |
| **P14 (737/972)** | Forward: 5′-GTCAATTGCATAAGCCTTCAGGGAAAG-3′<br>Reverse: 5′-TTGCGGCCGCCTAAAGAGAAAAGAGATATG-3′ |
| **P15 (850/972)** | Forward: 5′-GTCAATTGTATACGAATCCAGACAAG-3′<br>Reverse: 5′-TTGCGGCCGCCTAAAGAGAAAAGAGATATG-3′ |
| ***TARDBP* -721/intron1 (-721/+1094, total 1815 nucleotides)** | |
| **P16** | Forward: 5′-GTCAATTGCGCCTACCGCGTTCAAGCAATTC-3′<br>Reverse: 5′-TTGCGGCCGCCTAAAGAGAAAAGAGATATG-3′ |
| **P16 (Δexon 1)** | Forward: 5′-GTCAATTGCGCCTACCGCGTTCAAGCAATTC-3′<br>Reverse: 5′-TTGCGGCCGCGGCGTCCTCTCCCACCGGTTG-3′<br>Forward: 5′-TTGCGGCCGCGTGAGTCGCGGAGCCTTC-3′<br>Reverse: 5′-TTGCGGCCGCCTAAAGAGAAAAGAGATATG-3′ |
| **P16 (Δintron 1)** | Forward: 5′-GTCAATTGCGCCTACCGCGTTCAAGCAATTC-3′<br>Reverse: 5′-TTGCGGCCGCCCGCTAGGCCGCTGCTGG-3′ |
| ***TARDBP* intron2 (+1345/+4222, total 2878 nucleotides)** | |
| **P3 (+1345/+4222)** | Forward: 5′-GCCAATTGGTTTGTTACCATTTGG-3′<br>Reverse: 5′-TTGCGGCCGCCTAAATAAAACGAGCAG-3′ |
| **Luciferase reporter** | |
| **Luc2** | Forward: 5′-ATTTGCGGCCGCCCACCATGGAAGATGCCAAAAAC-3′<br>Reverse: 5′-GGGGTACCTTACACGGCGATCTTGCC-3′ |

**Table S4. Primers used for RT-PCR analysis.**

| | |
|---|---|
| ***TARDBP* exon 1 to Luc2** | Forward: 5′-CAAGCTTGGTGGGCGGGGGGAGGAG-3′<br>Reverse: 5′-CTTCATAGCTTCTGCCAGCC-3′ |
| **Endogenous TDP-43** | Forward: 5′-GTTACAGCCCAGTTTCCAG-3′<br>Reverse: 5′-CAGTCATGTCCTCTGTACA-3′ |
| **ACTB** | Forward: 5′-TCACCATGGATGATGATATC-3′<br>Reverse: 5′-CTGGGTCATCTTCTCGCGG-3′ |

**Table S5. Primers used for ChIP assay.**

| | |
|---|---|
| ***TARDBP* -721 to -431** | Forward: 5′-GTCAATTGCGCCTACCGCGTTCAAGCAATTC-3′<br>Reverse: 5′-GAGAGTAGCACAGAGTCTCG-3′ |
